# Identification and Characterization of a Red-Light Sensor FphA in *Aspergillus flavus*

**DOI:** 10.3390/ijms27062621

**Published:** 2026-03-13

**Authors:** Kunzhi Jia, Qianhua Zeng, Shuqi Huang, Fufa Tong, Jingwen Huang, Shihua Wang

**Affiliations:** Key Laboratory of Pathogenic Fungi and Mycotoxins of Fujian Province, Key Laboratory of Biopesticide and Chemical Biology of Education Ministry, School of Life Sciences, Fujian Agriculture and Forestry University, Fuzhou 350002, China; kjia@fafu.edu.cn (K.J.);

**Keywords:** FphA, conidiation, aflatoxin biosynthesis, red light

## Abstract

*Aspergillus flavus* (*A. flavus*) is a common contaminant of food and feed due to the production of aflatoxin B_1_, which is susceptible to environmental signals. Nevertheless, how red light plays a role in *A. flavus* remains unclear. Here, we identified the uncharacterized hypothetical protein G4B84_010091 as a red-light sensor, defined as fungal phytochrome A (FphA), in *A. flavus*. The *fphA* knockout strain (Δ*fphA*) and complementary strain (*fphA*-com) were successfully constructed to characterize the function of FphA. Our results indicated that aflatoxin B_1_ biosynthesis was promoted, while the development of conidia and sclerotia as well as the infection of peanuts were impaired in Δ*fphA* when compared with WT or *fphA*-com. The FphA^ΔRR^ domain deletion mutant exhibited all the phenotypes observed in the Δ*fphA* strain, indicating that the RR domain is indispensable for the function of FphA. In summary, FphA is involved not only in the formation of spores and sclerotia, but also in aflatoxin B_1_ biosynthesis and the pathogenicity of *A. flavus*, which offers a potential target for novel approaches to controlling the dispersal and toxin production of this fungus.

## 1. Introduction

*Aspergillus flavus* (*A. flavus*) can widely contaminate various food and feed sources around the world [1]. As a ubiquitous filamentous fungus, 30–60% of *A. flavus* strains are able to produce the class I carcinogen B-type aflatoxins (AFs) [2], which usually cause substantial economic losses in agriculture and pose significant threats to human and animal health [3]. Therefore, understanding the biological processes of *A. flavus* is crucial for developing effective strategies to reduce aflatoxin contamination. During the past decade, scientists have conducted intensive studies on *A. flavus*. Zhang et al. [4] reported that water activity played a role in growth and AF biosynthesis in *A. flavus*. Gallo et al. [5,6] proved that temperature and water activity were both essential for aflatoxin B_1_ (AFB_1_) biosynthesis. Extracellular pH conditions also significantly affect AF production in *A. flavus* [7,8]. All these studies suggest that AF biosynthesis is closely related to environmental factors. In *A. flavus*, AF biosynthesis is mainly controlled by the AF gene cluster on chromosome 3. The AF gene cluster includes 30 genes and is regulated by AflR and AflS [7]. AflR act as a positive regulator of the AF gene cluster, including *aflR* itself. AflS usually interacts with AflR and co-activates the AF gene cluster, regulating AF production [7].

During the growth and storage of food such as peanuts and maize, which are easily contaminated by *A. flavus* [1], light is an unavoidable environmental factor. In *Aspergillus nidulans*, photoreceptors such as LreA and phytochrome A mediate the effects of blue and red light, respectively, which play essential roles in growth and secondary metabolism [9]. By converting physical signals into biological information, phytochrome A regulates a wide range of physiological processes, including photomorphogenesis, pigment synthesis, and circadian rhythms [10,11]. Moreover, phytochrome A is widely found in plants, fungi, and bacteria [12]. In plants, phytochrome A can perceive red light and initiate conformational changes, which activate the light signaling pathway through a series of phosphorylation events [13].

Canonical phytochrome A contains a tridomain composed of PAS (Per-Arnt-Sim), GAF (cGMP phosphodiesterase, adenylyl cyclase, and FhlA protein), and PHY (phytochrome-specific) at the N-terminus, which is known as the photosensory module responsible for binding to a bilin chromophore and perceiving light [14,15]. The C-terminal output module typically includes HK (histidine kinase) domain, which is responsible for dimerization and signal transmission to downstream targets [10,12,15]. Interestingly, fungal phytochrome A (FphA) has an additional RR (response regulator) domain at the C-terminus, which is believed to be involved in phosphorylation transfer and protein interaction [12,13,16]. RR knockout mutants exhibited reduced responses to red light [12]. Thus, phytochrome A, composed of photosensory and output modules, plays a role in various biological processes in response to red light in plants and fungi.

The physiological function of red-light sensors remains unknown in *A. flavus*, which is a fungus of concern in agriculture. Our study aims to identify the receptor for red light in *A. flavus* and further characterize the function of this light receptor in conidiation and secondary metabolism, which would provide valuable insights into the light-dependent behaviors and adaptations of *A. flavus*.

## 2. Results

### 2.1. Identification of FphA as a Red-Light Sensor in A. flavus

**Figure 1:**
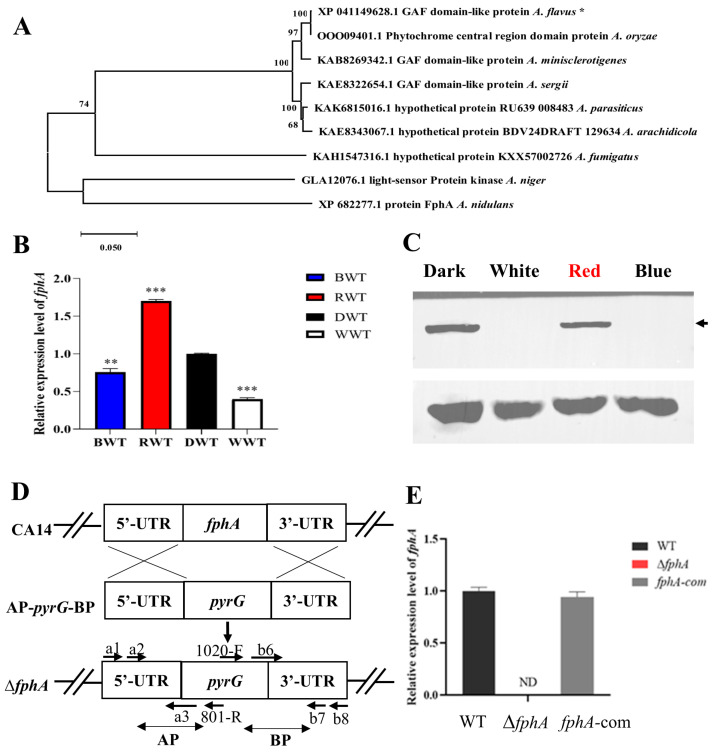
FphA responds to red light in *Aspergillus flavus* (*A. flavus*). (**A**) Phylogenetic tree of FphA homologous proteins from *A. flavus*, *Aspergillus oryzae*, *Aspergillus minisclerotigenes*, *Aspergillus sergii*, *Aspergillus parasiticus*, *Aspergillus arachidicola*, *A. fumigatus*, *Aspergillus niger*, and *A. nidulans*. The numbers at the nodes represent bootstrap values. The asterisk indicates the strain investigated in this study. (**B**) RNA expression of *fphA* in WT *A. flavus* under various light conditions for a continuous 72 h. BWT represents WT strains cultured in blue LED light (450 nm, 3.7 μmol/(m^2^·s)). Similarly, RWT, DWT, and WWT represent WT strains cultured in red LED light (700–710 nm, 15.2 μmol/(m^2^·s)), dark (0 μmol/(m^2^·s)), and white LED light (14.2 μmol/(m^2^·s)), respectively. (**C**) The protein level of FphA was determined in the *fphA-HA* mutant strain cultured in dark, white, red, or blue light for 72 h. The expression of *fphA* was determined by Western blot using the anti-HA antibody (upper panel). The arrow points to the target band of FphA. Beta-actin was used as the loading control, shown in the lower panel. (**D**) A typical schematic description of *fphA* disruption. UTR, untranslation region. AP and BP represent the A and B homology arm parts, respectively. (**E**) Expression of *fphA* was determined by RT-qPCR in different *A. flavus* strains. ND, not detectable. (**, *p* < 0.01; ***, *p* < 0.001).

Based on a BLAST search using the FphA protein sequence from *A. nidulans* (XP_682277.1), an uncharacterized protein, G4B84_010091 (XP_041149628.1), was identified as a red-light sensor and defined as FphA in *A. flavus*. The phylogenetic tree was constructed using 10 homologous protein sequences from various fungal species. As shown in Figure 1A, FphA of *A. flavus* was highly conserved in the genus *Aspergillus* and was very similar phylogenetically to that of *A. oryzae* (98.4% similarity). To investigate the relationship between light and FphA, the expression of FphA under different light conditions was examined. As shown in Figure 1B, the transcripts of *fphA* were induced 1.6-fold by red light compared with those in the dark. The transcripts of *fphA* were also decreased by 25% under blue light compared with those in the dark. Correspondingly, FphA protein was highly expressed in the dark and under red light (Figure 1C). To further investigate the function of FphA in *A. flavus*, the *fphA* knockout strain (Δ*fphA*) and complementary strain (*fphA*-com) were constructed using the strategy shown in Figure 1D. The mutant strains were verified by PCR and DNA sequencing (Figure S1). Expression levels of *fphA* were further checked by RT-qPCR. As shown in Figure 1E, the expression of *fphA* was not detected in Δ*fphA*, indicating that the *fphA* mutant strains were successfully constructed.

### 2.2. FphA Absence Impaired Conidia Development

**Figure 2:**
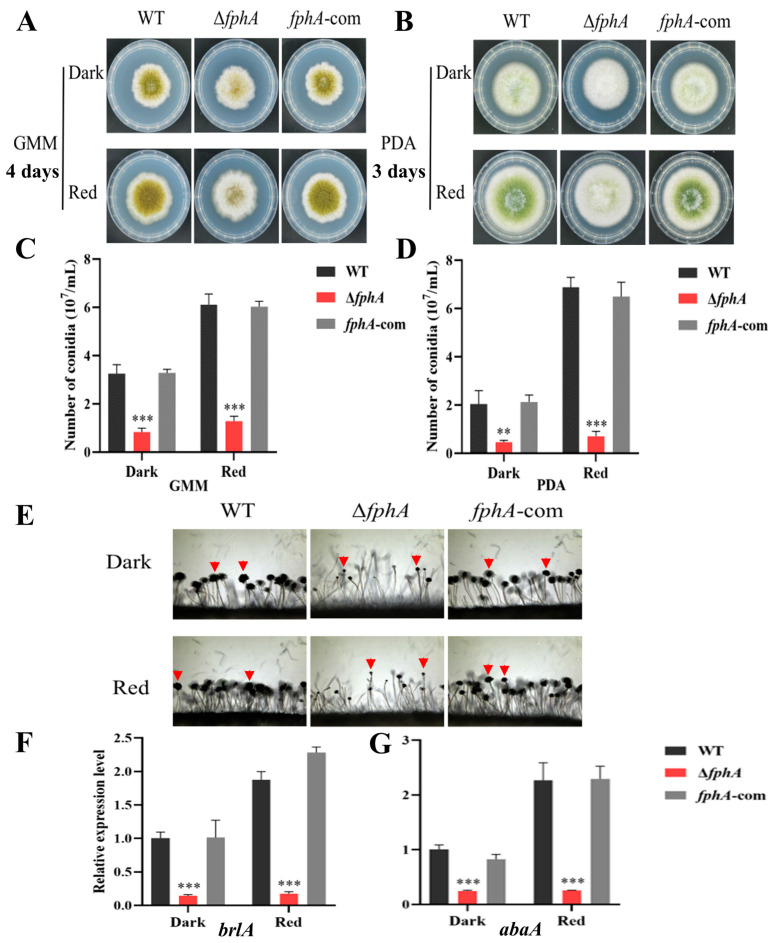
FphA regulates the conidia development of *A. flavus* in the dark and red LED light (700–710 nm, 15.2 μmol/(m^2^·s)). (**A**,**B**) The growth morphology in WT, Δf*phA*, and *fphA*-com grown for 3–4 days on GMM and PDA, respectively. (**C**,**D**) Comparison of conidia numbers in WT, Δ*fphA*, and *fphA*-com shown in (**A**,**B**). (**E**) Conidiophore morphology of WT, Δ*fphA*, and *fphA*-com. Red triangles indicate representative examples. (**F**,**G**) The expression of *brlA* and *abaA* in WT, Δ*fphA*, and *fphA*-com. (**, *p* < 0.01; ***, *p* < 0.001).

The colony diameter of Δ*fphA* is equal to that of WT and *fphA*-com regardless of culture condition or light signal, showing that FphA has no significant effect on the growth of *A. flavus* (Figure 2A,B). However, the growth phenotype of Δ*fphA* is observably different from the control strains, which hints at a potential role of FphA in the conidiation of *A. flavus*. To study the effect of FphA on conidia development, the conidia number was compared between Δ*fphA* and WT or *fphA*-com. As shown in Figure 2C,D, compared with the dark condition, the conidia were increased in WT or *fphA*-com under red light, suggesting that red light was able to induce conidia development. This increase disappeared in Δ*fphA*, suggesting that FphA mediates the promotion of conidia development induced by red light. Poor conidiophore development was further observed in Δ*fphA* (Figure 2E). The expression levels of *brlA* and *abaA*, which are the master regulators of conidiation, were significantly decreased in Δ*fphA* compared with WT and *fphA*-com (Figure 2F,G). These results indicated that the absence of FphA inhibited the transcription of *brlA* and *abaA*, which impaired conidiophore development and conidiation.

### 2.3. FphA Plays a Role in the Sclerotia of A. flavus

**Figure 3:**
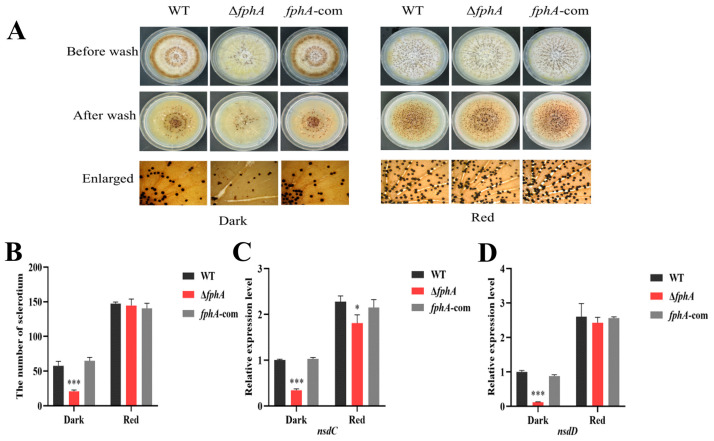
FphA regulates the sclerotia development of *A. flavus*. (**A**) Sclerotia formation in WT, Δ*fphA*, and *fphA*-com. The left panel represents *A. flavus* grown in the dark (0 μmol/(m^2^·s)) for 8 days, and the right panel represents *A. flavus grown* in red light (700–710 nm, 15.2 μmol/(m^2^·s)) for 8 days. (**B**) Comparison of sclerotia numbers in Δ*fphA* and *fphA*-com. (**C**,**D**) The expression of *nsdC* and *nsdD* in the different *A. flavus* strains above. (*, *p* < 0.05; ***, *p* < 0.001).

The sclerotia were decreased in Δ*fphA* when compared to the WT and *fphA*-com strains in the dark (Figure 3A,B). Correspondingly, the expression levels of *nsdC* and *nsdD*, which are the key genes for sclerotia development, were significantly downregulated in Δ*fphA* grown in the dark (Figure 3C,D). In contrast, there was no significant difference in *nsdC* and *nsdD* expression between Δ*fphA* and the control strains (WT and *fphA*-com) in the red light, which is consistent with the observation of equal sclerotia in different *fphA* strains in the red light (Figure 3). The above results indicated that the absence of FphA reduces the ability of *A. flavus* to produce sclerotia in the dark. Also, the number of sclerotia of WT *A. flavus* grown in red light was 2.7 times that grown in the dark, suggesting that red light is favorable for sclerotia development. In contrast, the number of sclerotia of Δ*fphA* in red light was seven times that in the dark (Figure 3B). Correspondingly, consistent changes were observed in the expression of *nsdC* and *nsdD* (Figure 3C,D).

### 2.4. The Effect of FphA on the Biosynthesis of Aflatoxins

**Figure 4:**
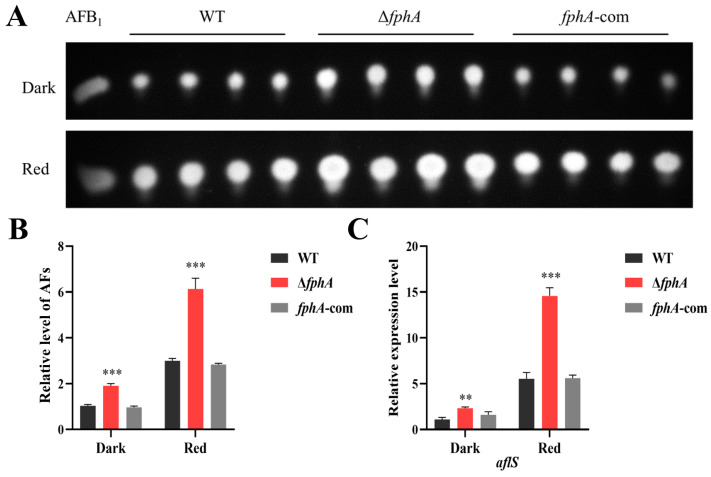
The effect of FphA on AF biosynthesis. *A. flavus* were cultured in the dark (0 μmol/(m^2^·s)) or under red light (700–710 nm, 15.2 μmol/(m^2^·s)) for 5 days. (**A**) TLC chromatography of AFs synthesized in WT, Δ*fphA*, and *fphA*-com strains. The whole image of the TLC plate is provided in Figure S3. (**B**) Relative quantitative analysis of AFs. (**C**) Relative expression levels of the *aflS* gene in different strains. (**, *p* < 0.01; ***, *p* < 0.001).

To study the effect of red light on AF biosynthesis, the AF level was determined in the dark and red light, respectively. As shown in Figure 4A,B, the production of AFs was significantly induced by red light in WT, Δ*fphA*, and *fphA*-com, which was related to the upregulation of *aflS*, a regulator that positively controls AF biosynthesis (Figure 4C). To investigate the role of FphA in the regulation of red light on AF biosynthesis, the AF level was compared in WT, Δ*fphA*, and *fphA*-com in the dark and red light. As shown in Figure 4A, the AF level was significantly increased in Δ*fphA* when compared with that in WT and *fphA*-com regardless of the light conditions (Figure 4A,B). The transcriptional expression levels of *aflS* [17,18] were significantly upregulated in Δ*fphA* (Figure 4C), suggesting that FphA negatively regulates AF biosynthesis by inhibiting the transcription of *flS*. The expression of *velvetA* (*veA*) was also observed in Δ*fphA* (Figure S2), suggesting that VeA was involved in the regulation of AF biosynthesis in Δ*fphA*.

### 2.5. The Effect of FphA on the Pathogenicity of A. flavus

**Figure 5:**
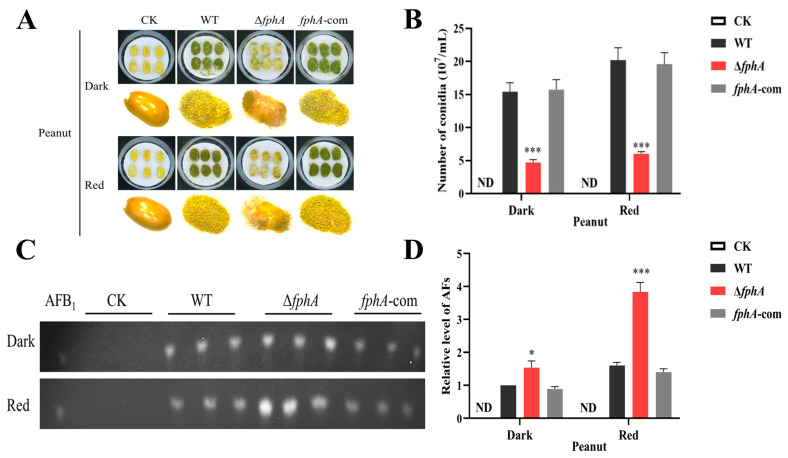
The effect of FphA on the pathogenicity of *A. flavus* in the dark or red light (700–710 nm, 15.2 μmol/(m^2^·s)) for 7 days. (**A**) The morphology of peanuts infected by WT, Δ*fphA*, and *fphA*-com of *A. flavus*. (**B**) Comparison of conidia numbers in peanuts infected with WT, Δ*fphA*, and *fphA*-com strains. (**C**) TLC chromatography of AFs in peanuts infected with WT, Δ*fphA*, and *fphA*-com strains. (**D**) AF levels from peanuts infected with WT, Δ*fphA*, and *fphA*-com as in C. ND, not detectable. (*, *p* < 0.05; ***, *p* < 0.001).

Peanut infection was used to study the pathogenicity of *A. flavus* strains. As shown in Figure 5A,B, poor growth and fewer conidia were observed in Δ*fphA* on peanuts when compared to WT or *fphA*-com. Notably, the number of conidia in Δ*fphA*-infected peanuts decreased by two-thirds compared with that of the control strains (Figure 5B), which has great significance for agriculture. Similar results were also observed in infected maize (Figure S4). Considering the toxicity of AFs, the AF level was also determined. As shown in Figure 5C,D, the AF level was increased in Δ*fphA*-infected peanuts, further indicating that FphA repressed AF biosynthesis. These results showed that the absence of FphA impaired the pathogenicity of *A. flavus* to oil seeds, which are important sources of agricultural products. At the same time, red light exhibited a beneficial effect on the pathogenicity of *A. flavus* in WT, Δ*fphA*, and *fphA*-com (Figure 5A,B). All these results suggest that FphA plays important roles in the pathogenicity of *A. flavus*.

### 2.6. The Response Regulator (RR) Domain Is Vital for the Function of FphA

**Figure 6:**
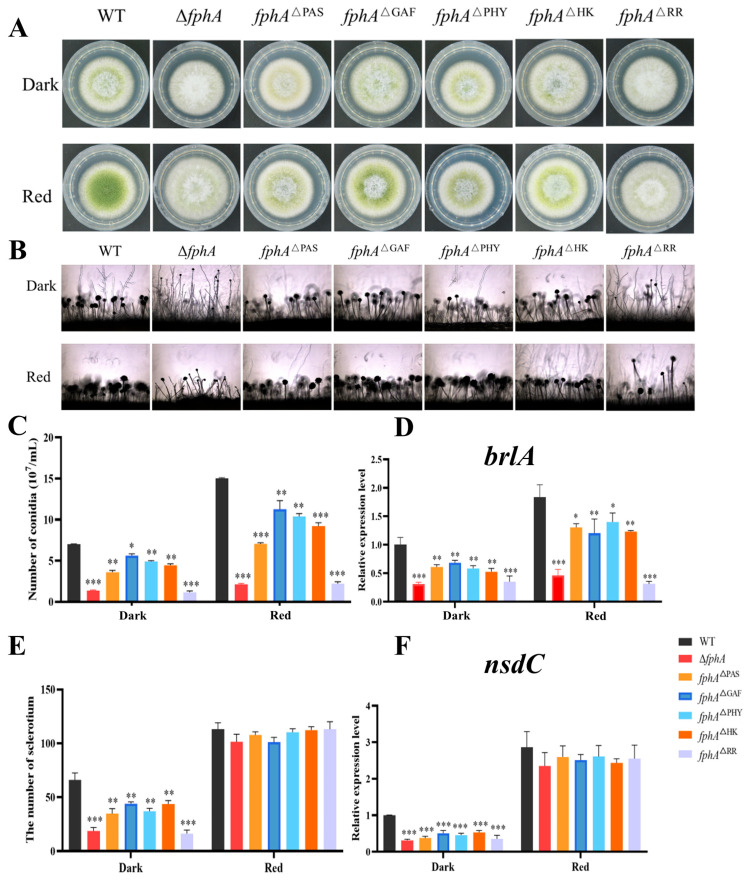
The conidiation and sclerotia development of WT and mutant strains (Δ*fphA*, *fphA*^ΔPAS^, *fphA*^ΔGAF^, *fphA*^ΔPHY^, *fphA*^ΔHK^, and *fphA*^ΔRR^) in the dark or red light (700–710 nm, 15.2 μmol/(m^2^·s)). (**A**) Growth morphology of WT and mutant strains grown for 3 days. (**B**) Conidia development of WT and mutant strains grown for 3 days. (**C**) The conidia number of WT and mutant strains grown for 3 days. (**D**) The relative expression of *brlA* in WT and mutant strains grown for 3 days. (**E**) The sclerotia number of WT and mutant strains grown for 8 days. (**F**) The relative expression of *nsdC* in WT and mutant strains grown for 8 days. (*, *p* < 0.05; **, *p* < 0.01; ***, *p* < 0.001).

To study the function of FphA domains, five mutants for domain deletion (*fphA*^ΔPAS^, *fphA*^ΔGAF^, *fphA*^ΔPHY^, *fphA*^ΔHK^, and *fphA*^ΔRR^) were constructed. As shown in Figure 6A, the RR domain mutant (*fphA*^ΔRR^) exhibited a similar phenotype to Δ*fphA*. Conidiophore development of *fphA*^ΔRR^ resembled the phenotype of Δ*fphA* (Figure 6B), which confirmed that the RR domain is critical for the function of FphA in conidiation. Correspondingly, conidia were decreased in all the domain deletion mutants compared with those in WT, indicating that the absence of FphA or its conserved domains impaired conidia development (Figure 6C). This impairment is highly related to the decreased expression of *brlA* (Figure 6D), which is a master regulator at the early stage of conidiation [19]. These results demonstrated that the conserved domains contributed to the effect of FphA on conidiation, and the RR domain is vital for the function of FphA. Sclerotia were decreased in the dark in Δ*fphA* and the domain deletion mutants when compared to WT, which is consistent with the decreased expression of *nsdC* in Δ*fphA* and the domain deletion mutants (Figure 6E,F), implying that the RR domain and other conserved domains contributed to the effect of FphA on sclerotia formation in the dark by affecting the expression of *nsdC*.

**Figure 7:**
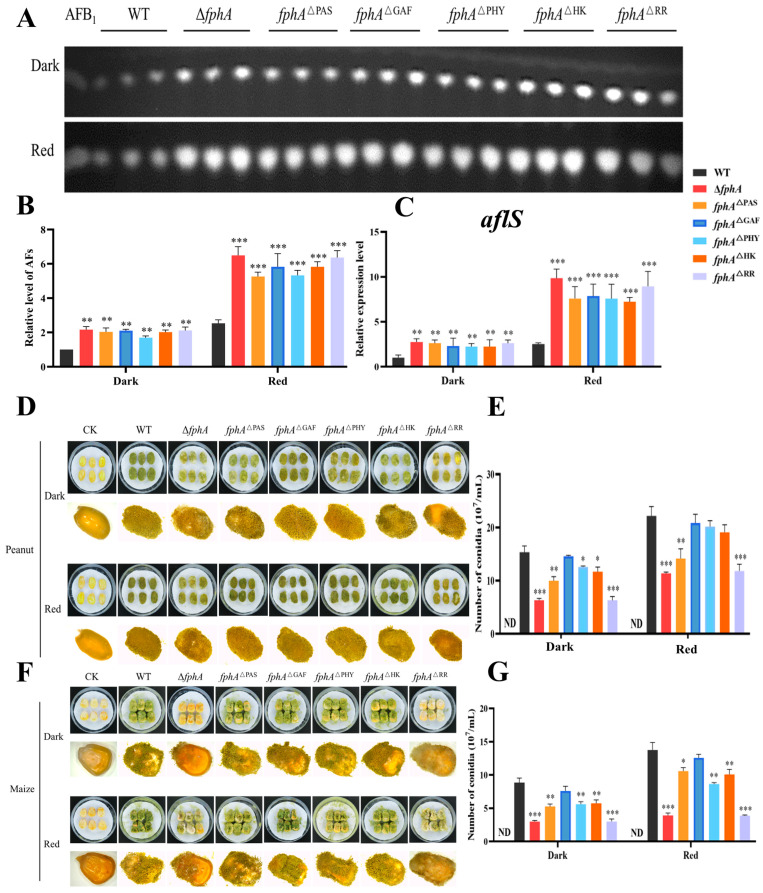
The AF levels and pathogenicity of WT and mutant strains (Δ*fphA*, *fphA*^ΔPAS^, *fphA*^ΔGAF^, *fphA*^ΔPHY^, *fphA*^ΔHK^, and *fphA*^ΔRR^) in the dark or red light (700–710 nm, 15.2 μmol/(m^2^·s)). (**A**) TLC analysis of AFs in WT and mutant strains grown for 5 days. (**B**) The relative quantification of AFs in WT and mutant strains. (**C**) *aflS* expression in WT and mutant strains for 5 days. (**D**) Peanuts infected with WT and mutant strains grown for 7 days. (**E**) Conidia number of WT and mutant strains as in (**D**). (**F**) Maize infected with WT and mutant strains for 7 days. (**G**) Conidia number of WT and mutant strains as in (**F**). (*, *p* < 0.05; **, *p* < 0.01; ***, *p* < 0.001).

Compared with WT, the AF level was significantly increased (Figure 7A,B), and the expression of *aflS* involved in AF biosynthesis was upregulated in all the domain deletion mutants (Figure 7C). These phenomena were equal to those in Δ*fphA*, suggesting that the intact protein structure is important for the function of FphA in AF biosynthesis, and that any domain loss significantly impairs the effect of FphA on *aflS* expression and AF production in *A. flavus* (Figure 7C). Furthermore, infections were impaired in peanuts and maize with the domain deletion mutants as well as Δ*fphA* (Figure 7D–F). Correspondingly, conidia were decreased in all the domain deletion mutants as well as Δ*fphA* on peanuts and maize (Figure 7E–G). Remarkably, the mutant *fphA*^ΔRR^ exhibits almost the same phenotype as Δ*fphA* in the observed phenotypes shown in Figure 6 and Figure 7, suggesting that the RR domain is vital for the function of FphA.

## 3. Discussion

FphA can sense red light signals and play a role in the developmental process based on studies of *A. nidulans* [20]. However, whether *A. flavus* has a light receptor remains largely unknown. In this study, we identified a red-light receptor defined as FphA in *A. flavus* for the first time. Moreover, FphA was shown to be induced by red light (Figure 1B,C), which will be beneficial for amplifying the effect of red light. The RNA level of *fphA* was significantly induced after culture for 72 h in red light (Figure 1B), suggesting that the induction is attributed to the late effect of red light. The upregulation of FphA shown in Figure 1C may also partly be due to the decreased degradation of FphA in red light observed before [10,21]. Considering the high level of FphA in the dark (Figure 1B,C), the expression of FphA is probably more sensitive to wavelength than to light flux density. Due to the potential toxic effect of blue light on cellular growth [9,22,23,24], a control dosage of blue light was not used. Even under blue light of comparatively low intensity (Figure 1B,C), it was not beneficial to the expression of FphA. To study the function of FphA, Δ*fphA*, and *fphA*-com were successfully constructed using the strategy described in Figure 1, proving to be effective for inactivating genes of various sizes in *A. flavus* [21,24].

Our study demonstrated that the absence of FphA impaired conidiation (Figure 2A,B) by downregulating *brlA* and *abaA* expression. Impaired conidiation was also observed in Δ*fphA* of *A. nidulans* [25]. Considering that BrlA and AbaA are the master regulators for conidia development [19,26], the expression of *brlA* and *abaA* is crucial for conidiation. Our study revealed that the critical step of conidia development is regulated by FphA in *A. flavus*. Impaired expression of *brlA* and *abaA* subsequently caused the poor conidiophore and conidiation observed in Figure 2, which has also been reported previously in *A. flavus* [24]. Our results also revealed that FphA promoted sclerotia by upregulating the expression of *nsdC* and *nsdD* in the dark (Figure 3). Interestingly, this promotion disappeared in red light, suggesting that FphA is helpful but not necessary for sclerotia formation in *A. flavus* grown in red light (Figure 3).

Our study indicated that FphA negatively regulates AF biosynthesis in *A. flavus*, consistent with the finding that FphA represses the formation of sterigmatocystin (ST), the precursor of AFs including AFB_1_ [7], in *A. nidulans* [25]. Accordingly, AF levels were increased in Δ*fphA* compared with WT and *fphA*-com. Although there are various types of aflatoxins, AFB_1_ appears to be the predominant aflatoxin (>80%) [27]. The results of TLC were always highly correlated with HPLC in *A. flavus* [27]. Thus, the results of TLC probably represent the level of AFB_1_, considering there was only one band on the TLC plate (Figure S3). We further demonstrated that the increase in AFs was consistent with the upregulation of *aflS* (Figure 4B,C), which is required for AF biosynthesis [7,28]. Overexpression of *aflS* resulted in an increase in AFs and pathway intermediates in *A. flavus* [17]. Disruption of *aflS* in *A. parasiticus* resulted in a reduction of some genes related to AFs and AF intermediates [18]. Interestingly, red light has a positive role in AF biosynthesis in *A. flavus*, which is consistent with the effect of red light on ST formation in *A. nidulans* [25]. At the same time, red light promotes AF biosynthesis regardless of *fphA* status, suggesting that other potential receptors also mediate the effect of red light, which agrees with the finding that phytochromes other than phytochrome A can sense red light in plants [29]. VeA protein plays an important role in secondary metabolism in fungi [30]. Notably, VeA has a positive effect on AFB_1_ production in *A. flavus* [30]. Thus, the increased expression of *veA* promoted AF biosynthesis in Δ*fphA*, consistent with the observation in Figure 4B. On the other hand, FphA, which directly interacts with VeA [30], may interfere with the function of VeA in AF biosynthesis. Considering the presence of other phytochromes, this interference of FphA together with other red-light sensors may achieve the maximum effect in red light. Thus, the synthesis of AFs was induced in Δ*fphA*, as well as WT and *fphA*-com.

Poor growth and fewer conidia were observed in Δ*fphA* on peanuts, suggesting that FphA contributes to the pathogenicity of *A. flavus* regardless of the light condition (Figure 5A,B), which agrees with the finding that disruption of a phytochrome-like gene significantly reduces the pathogenicity of *Botrytis cinerea* [31]. Phytochrome knockout mutants of *Agrobacterium fabrum* diminished plant infection [32]. At the same time, the AF level was significantly increased in Δ*fphA* compared with WT and *fphA*-com (Figure 5C,D), consistent with the data observed in culture medium (Figure 4). Considering the toxicity of AFs [33], peanuts infected with Δ*fphA* remain hazardous to the public and animals. Thus, red light contributes to the pathogenicity and virulence of *A. flavus*, in which FphA possibly plays a vital role (Figure 5), consistent with observations in *Botrytis cinerea* [31].

Our results also found that the absence of the conserved domains including PAS, GAF, PHY, HK, and RR of FphA impaired conidiophore and conidia development. Given that PAS, GAF, and PHY possibly constitute the photosensory module [10,15], the loss of FphA function in conidia development is partly related to the impaired light signal in *fphA*^ΔPAS^, *fphA*^ΔGAF^, and *fphA*^ΔPHY^ (Figure 6). In contrast, FphA inhibited AF biosynthesis by repressing the expression of *aflS*, in which the domains including PAS, GAF, PHY, HK, and RR were all necessary for the role of FphA in AF biosynthesis (Figure 7A–C). The absence of FphA domains impaired the pathogenicity of *A. flavus* to varying degrees, resembling the phenotype of the Δ*fphA* strain (Figure 7D–G). Therefore, the PAS, GAF, PHY, HK, and RR domains in FphA all play important roles in spore production, sclerotia formation, AF biosynthesis, and infection ability. The phenotype of the *fphA*^ΔRR^ strain is consistent with that of the Δ*fphA* strain (Figure 6 and Figure 7), suggesting that the RR domain plays a critical role in the intact function of FphA. Given that the RR domain was involved in the phosphorylation transfer and protein interaction [12,13,16], it is reasonable that the function of FphA was related to the signal transmission of red light, especially concerning the RR domain in *A. flavus*.

In conclusion, a red-light sensor defined as FphA has been identified in *A. flavus*, which plays a role not only in the formation of spores and sclerotia, but also in AF biosynthesis and the pathogenicity of *A. flavus*. The RR domain is found to be vital for the intact function of FphA. Our findings offer potential targets for novel approaches to control and prevent *A. flavus*.

## 4. Materials and Methods

### 4.1. Strains and Light Source

All *A. flavus* strains used in this study are listed in Table 1. Red LED light (700–710 nm), blue LED light (450 nm), and white LED light were used as the light sources for the growth of *A. flavus*. The photon flux density, as marked above, was measured using a digital PAR Meter named AH-Quantuv (Fuhongdi Technology Co., Ltd., Zhongshan, China).

### 4.2. Phylogenetic Tree Building

Homologous proteins of FphA from various *Aspergillus* species, including *A. flavus*, *Aspergillus oryzae*, *Aspergillus minisclerotigenes*, *Aspergillus sergii*, *Aspergillus parasiticus*, *Aspergillus arachidicola, A. fumigatus*, and *Aspergillus niger*, were obtained by BLAST (version 2.16.0+) with the FphA sequence (XP_682277.1) from *A. nidulans* in NCBI. After the complete protein sequences were downloaded, a phylogenetic tree was built using the neighbor-joining method with a bootstrap test of 1000 replicates within MEGA 7.0 software. Bootstrap values are shown at the nodes of the phylogenetic tree.

### 4.3. Construction of fphA Mutant Strains

The knockout strain (Δ*fphA*) and complementary strain (*fphA*-com) were constructed using the homology recombination method described previously [34]. In brief, the upstream (AP, A homology arm part) and downstream (BP, B homology arm part) sequences of the *fphA* gene, as well as the *pyrG* gene from *A. fumigatus*, were fused into an interruption fragment (AP-*pyrG*-BP) using a fusion PCR strategy. The AP fragment was amplified using primers *fphA*-A1 and *fphA*-A3 (Table A1), while the BP fragment was amplified using primers *fphA*-B6 and *fphA*-B8. The *pyrG* gene was amplified using primers *pyrG*-F and *pyrG*-R. *fphA*-A2 and *fphA*-B7 are nesting primers for further amplification of the AP-*pyrG*-BP fragment. The fused fragment (AP-*pyrG*-BP) was then transformed into *A. flavus* CA14 PTs protoplasts to construct the *fphA* knockout strain (Δ*fphA*). For the construction of the complementary strain (*fphA*-com), two steps of homology recombination were used. First, the *pyrG* gene in Δ*fphA* was replaced with the *fphA* gene fragment. Second, the *pyrG* gene was inserted into the site between the *fphA* ORF and the 3′UTR of the intermediate strain for the selection of the *fphA*-com strain. The positive strains were validated by PCR and further sequencing. Conserved domain deletion mutants including PAS (179–300 aa), GAF (378–559 aa), PHY (551–727 aa), HK (748–989 aa), and RR (1104–1232 aa) of FphA were constructed using the same strategy described above, and defined as *fphA*^ΔPAS^, *fphA*^ΔGAF^, *fphA*^ΔPHY^, *fphA*^ΔHK^, and *fphA*^ΔRR^, respectively.

### 4.4. Western Blot

*A. flavus* was cultured in the dark (0 μmol/(m^2^·s)), white LED light (14.2 μmol/(m^2^·s)), blue LED light (450 nm, 3.7 μmol/(m^2^·s)), or red LED light (700–710 nm, 15.2 μmol/(m^2^·s)) for a continuous 72 h. Then the mycelia were collected and completely freeze-dried, after which they were further crushed and lysed with RIPA (radio immunoprecipitation assay) buffer. The protein concentrations were determined with a BCA kit (Solarbio, Beijing, China). Equal amounts (30–50 μg) of proteins were resolved by 8% SDS-PAGE gels, transferred to a PVDF membrane, and probed with anti-HA antibodies [35]. Each experiment was repeated three times.

### 4.5. Analysis of Conidia and Sclerotia Production

Various strains of *A. flavus* were cultured on glucose minimal medium (GMM, containing 1.84 g/L ammonium tartrate, 0.52 g/L MgSO_4_·7H_2_O, 1.52 g/L KH_2_PO_4_, 10 g of glucose, 0.52 g/L KCl, and 1 mL trace elements) or potato dextrose agar (PDA) at 29 °C in the dark or red light (700–710 nm, 15.2 μmol/(m^2^·s)) for 3–4 days. The growth of *A. flavus* was photographed using a camera. For conidia quantification, the Petri dish was washed with 3 mL of 0.05% Tween-20 solution. Conidia were collected and counted on a hemocytometer. For analysis of sclerotia production, various strains of *A. flavus* were grown on complete medium (CM, containing 6 g/L yeast extract, 6 g/L peptone, and 3 g/L sucrose) at 37 °C in the dark or red light for 8 days. Then the sclerotia were counted manually under an optical microscope after the Petri dish was washed with 75% ethanol [36].

### 4.6. Aflatoxin Analysis

About 10^4^ spores were inoculated into 10 mL of YES (yeast extract with supplements) medium (150 g/L sucrose, 20 g/L yeast extract, 1 g/L MgSO_4_·7H_2_O), and cultured at 29 °C in continuous darkness or red light for 5 days. A total of 7 mL of post-cultivation medium was collected and then mixed with an equal volume of dichloromethane. After stirring at 150 rpm for 60 min and allowing the mixture to stand for 30 min, 5 mL of the lower layer was collected for further drying. The dried AFs were redissolved in 0.5 mL of dichloromethane for further thin-layer chromatography (TLC) analysis [37]. In brief, 5 µL of AF solution was loaded onto a silica gel plate and separated by chromatographic solution including acetone:dichloromethane (1:9, *v*/*v*). AFB_1_ (1 µg) was loaded as a standard. Silica gel plates were photographed with Gel DocXR+ (Bio-Rad, Hercules, CA, USA) under ultraviolet (UV) light at 365 nm. The relative quantification of AF amount was calculated relative to WT using GeneTools (version 4.03.05.0).

### 4.7. Infection of Peanuts and Maize Seeds

To assess the pathogenicity of *A. flavus*, peanut and maize seeds kept in our lab were washed with sodium hypochlorite, ethanol, and sterile water and then infected by immersion in a suspension of 10^7^ *A. flavus* spores for 30 min. The seeds were placed in Petri dishes lined with moist sterile filter paper for 7 days. The infected seeds were photographed and collected for spore counting and AF quantification [36].

### 4.8. RT-qPCR

Fungal spores were inoculated onto PDA and cultured for 72 h. The mycelia were scraped from the agar surface and ground into powder with liquid nitrogen. Total RNA was then extracted from the mycelia powder of *A. flavus* using an RNA Extraction Kit (TransGen, Beijing, China). First-strand cDNA was synthesized using TransScript RT/RI Enzyme Mix (TransGen, Beijing, China). RT-qPCR reactions were performed using SYBR Green Supermix (Vazyme Biotechnology, Nanjing, China) within the Applied Biosystems QuantStudio 1 Plus real-time PCR system (Applied Biosystems, Guangzhou, China). The 2^−ΔΔCT^ method was used to quantify the expression level of the target gene [38,39]. Primers for RT-qPCR are shown in Table A2.

### 4.9. Statistical Analysis

Data are presented as means ± SD. Statistical analysis was performed using GraphPad Prism 8 (GraphPad Software, San Diego, CA, USA), and one-way ANOVA followed by Tukey’s test was used. Differences were considered statistically significant when *p* < 0.05.

## Figures and Tables

**Table 1:** The *A. flavus* strains used in this study.

Strain Name	Genotype	Source
Wild-type	Δ*ku70*, Δ*niaD*, Δ*pyrG*::*pyrG*	Preserved in this laboratory
CA14 PTS	*∆ku70*, Δ*niaD*, ∆*pyrG*	Purchased from FGSC
Δ*fphA*	Δ*ku70*, Δ*niaD*, ∆*pyrG*, Δ*fphA*::*pyrG*	This study
*fphA*-com	Δ*ku70*, Δ*fphA*::*pyrG*, *fphA*::*pyrG*	This study
WT:3HA-FphA	Δ*ku70*, Δ*pyrG*, *fphA*::*pyrG*::3×HA	This study
*fphA* ^ΔPAS^	Δ*ku70*, Δ*niaD*, ∆*pyrG, fphA*^Δ^*^PAS^*::*pyrG*	This study
*fphA* ^Δ^ *^GAF^*	Δ*ku70*, Δ*niaD*, ∆*pyrG, fphA*^Δ^*^GAF^*::*pyrG*	This study
*fphA* ^Δ^ *^PHY^*	Δ*ku70*, Δ*niaD*, ∆*pyrG, fphA*^Δ^*^PHY^*::*pyrG*	This study
*fphA* ^Δ^ *^HK^*	Δ*ku70*, Δ*niaD*, ∆*pyrG, fphA*^Δ^*^HK^*::*pyrG*	This study
*fphA* ^Δ^ *^RR^*	Δ*ku70*, Δ*niaD*, ∆*pyrG, fphA*^Δ^*^RR^*::*pyrG*	This study

## Data Availability

The original contributions presented in this study are included in the article/Supplementary Material. Further inquiries can be directed to the corresponding author.

## Supplementary Materials

The following supporting information can be downloaded at: https://www.mdpi.com/article/10.3390/ijms27062621/s1.

## Appendix A

**Table A1:** PCR primers used in this study.

Primer	Sequence (5′ → 3′)	Characteristics
*fphA*-A1	CTATTGTCATCAGGGTTCCAGG	For Δ*fphA* upstream homology arm
*fphA*-A3	GAAGAGCATTGTTTGAGGCTTCAACGTCTTAATGACTGGAC	
*fphA*-B6	TCAGTGCCTCCTCTCAGACCGGCCCTGATCTCTGTGTTAA	For Δ*fphA* downstream homology arm
*fphA*-B8	CCCGTGTCTTGATTCCGATAG	
*pyrG*-F	GCCTCAAACAATGCTCTTCACCC	For *pyrG*
*pyrG*-R	GTCTGAGAGGAGGCACTGATGC	
*fphA*-A2	CGCCTATTATTTCGACGTA	*For* Δ*fphA overlap*
*fphA*-B7	GGTTACTCTTGCTCGTGGTG	
*fphA*-ORF-F	AGTGACAGCACGCTTGGTTTG	For *fphA* detection
*fphA*-ORF-R	TGATGAGATCCTGGCCCTTTT	
Test-F	GCCCCCAATCGTCAAGGTTT	For *pyrG* detection
Test-R	AGAGCGCCTTGAGAAAACCAGA	
*fphA*-SF	GTGTCACCTGTTGAGCGTG	For sequencing
*fphA*-SR	CGTCTCCTGTGGTAGATATCCT	
*fphA*-com-A1	TGGCCGCATTGTGTCACCTGTT	For *fph*A-com upstream arm
*fphA*-com-A3	GGGTGAAGAGCATTGTTTGAGGC- AGATTGCTTGGTGATTTTAAAAC	
*fphA*-com-B6	CGCATCAGTGCCTCCTCTCAGAC- ATAAAATCTTTACCAAAACATCC	For *fph*A-com downstream arm
*fphA*-com-B8	TACACCATCCAATCCCGTCCTC	
*fphA*-com-A2	ATGGAATCCAATGCTGACCCC	For nested PCR
*fphA*-com-B7	CTCAAAGCCCACCTCTTCCACA	
*gpda*-F	GAGCTCTGTACAGTGACCGGTGACT	For *gpda promoter*
*gpda*-R	GGTCAGCATTGGATTCCATTGTGATGTCTGCTCAAGCG	
*fphA*-A2-F	GTTCCCTTTTGCCCTCTCC	*fphA*-*HA* upstream homology arm
*fphA*-A2-R	CACCACTACCTCCGCCACCGCGCCCTTGAAACCAGCCA	
HA-*pyrG*-F	GGTGGCGGAGGTAGTGGTGGCGGAGGTTCAGGTGGCGGAGGTAGTTACC	*fphA*-*HA* downstream homology arm
HA-*pyrG*-R	GTCTGAGAGGAGGCACTGATGC	
FphA*-*HA-nesting-F	CCATGCCACGATAACGCTA	For nesting PCR
FphA*-*HA-nesting-R	AAAGCCCACCTCTTCCACA	
FphA*-*HA-SF	CTGCTCAATTTGATGCGGTC	For sequencing
FphA*-*HA-SR	AGAGCGCCTTGAGAAAACCAGA	

**Table A2:** Primers for RT-qPCR used in this study.

Primer	Sequence (5′ → 3′)	Characteristics
abaA-F	TCTTCGGTTGATGGATGATTTC	Primers for conidiation
abaA-R	CCGTTGGGAGGCTGGGT	
brlA-F	GCCTCCAGCGTCAACCTTC	
brlA-R	TCTCTTCAAATGCTCTTGCCTC	
nsdC-F	GCCAGACTTGCCAATCAC	Primers for sclerotia
nsdC-R	GGACTTGCGGGTCGTGCTA	
nsdD-F	GGACTTGCGGGTCGTGCTA	
nsdD-R	AGAACGCTGGGTCTGGTGC	
aflS-F	CGAGTCGCTCAGGCGCTCAA	Primers for AF biosynthesis
aflS-R	GCTCAGACTGACCGCCGCTC	
veA-F	ATCACCTTCGCATACAACGC	Primers for veA gene
veA-R	TCCAAGTAAGCCACTCCAGC	
Tubulin-F	GAAGGTGGAGGACATCTTG	Primers for reference gene
Tubulin-R	ACTCTTTCCGTGCCGTCTC	
