# Supplementary material for: Identification and Characterization of a Red-Light Sensor FphA in Aspergillus flavus
Source: Int J Mol Sci. 2026 Mar 13;27(6):2621. doi: 10.3390/ijms27062621 (PMC13027130; doi:10.3390/ijms27062621)
Supplement: Supplementary file 1 [file ijms-27-02621-s001.zip › ijms-4124092-supplementary.pdf]

Identification and Characterization of a Red-Light Sensor FphA in *Aspergillus flavus*

Kunzhi Jia †, Qianhua Zeng †, Shuqi Huang, Fufa Tong, Jingwen Huang, Shihua Wang*

Key Laboratory of Pathogenic Fungi and Mycotoxins of Fujian Province, Key Laboratory of Biopesticide and Chemical Biology of Education Ministry, School of Life Sciences, Fujian Agriculture and Forestry University, Fuzhou 350002, China

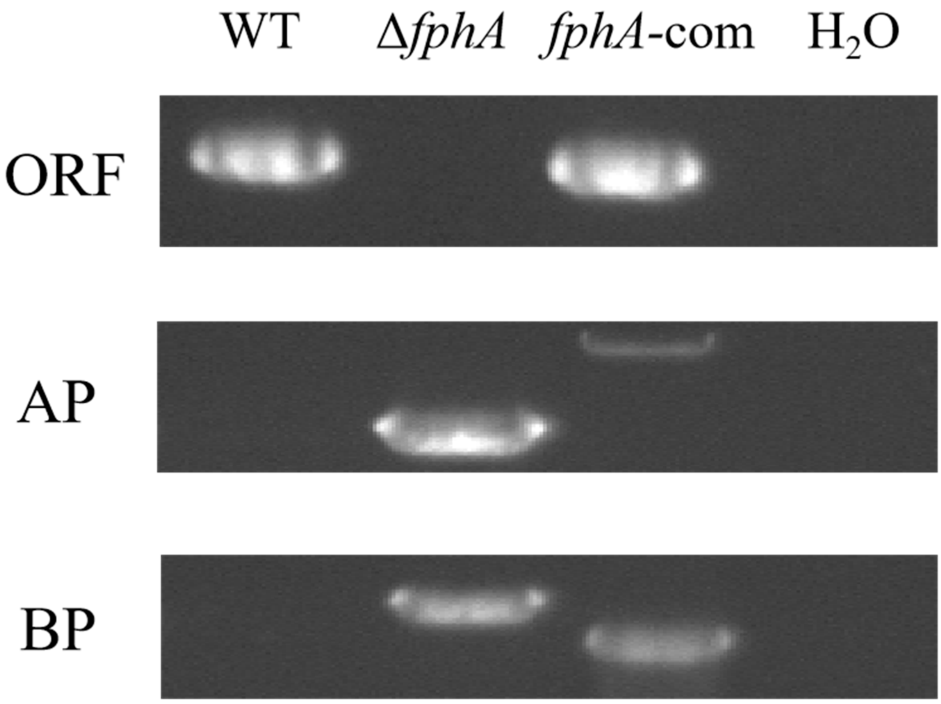

Supplementary Figure S1. PCR verification of WT, Δ*fphA*, and *fphA*-com. ORF represents open reading frame. AP and BP represent A and B homology arm part respectively.

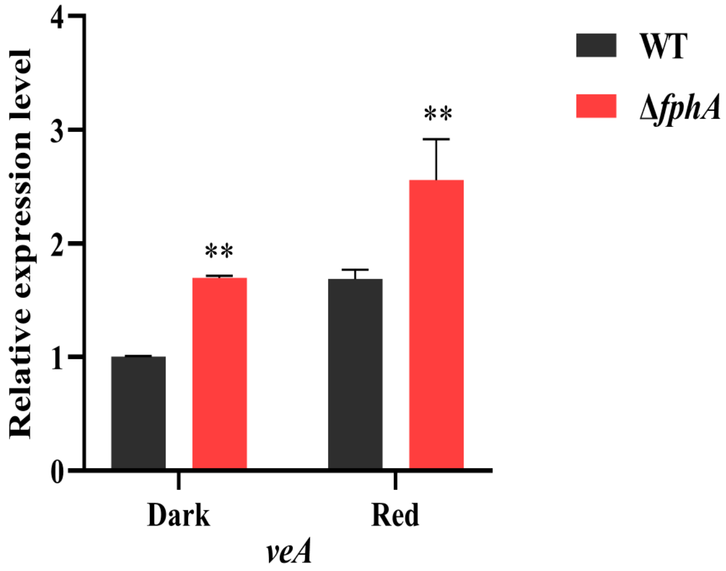

Supplementary Figure S2. Relative expression levels of *velvet A* (*veA*) gene in WT and Δ*fphA*. (**, $p<0.01$).

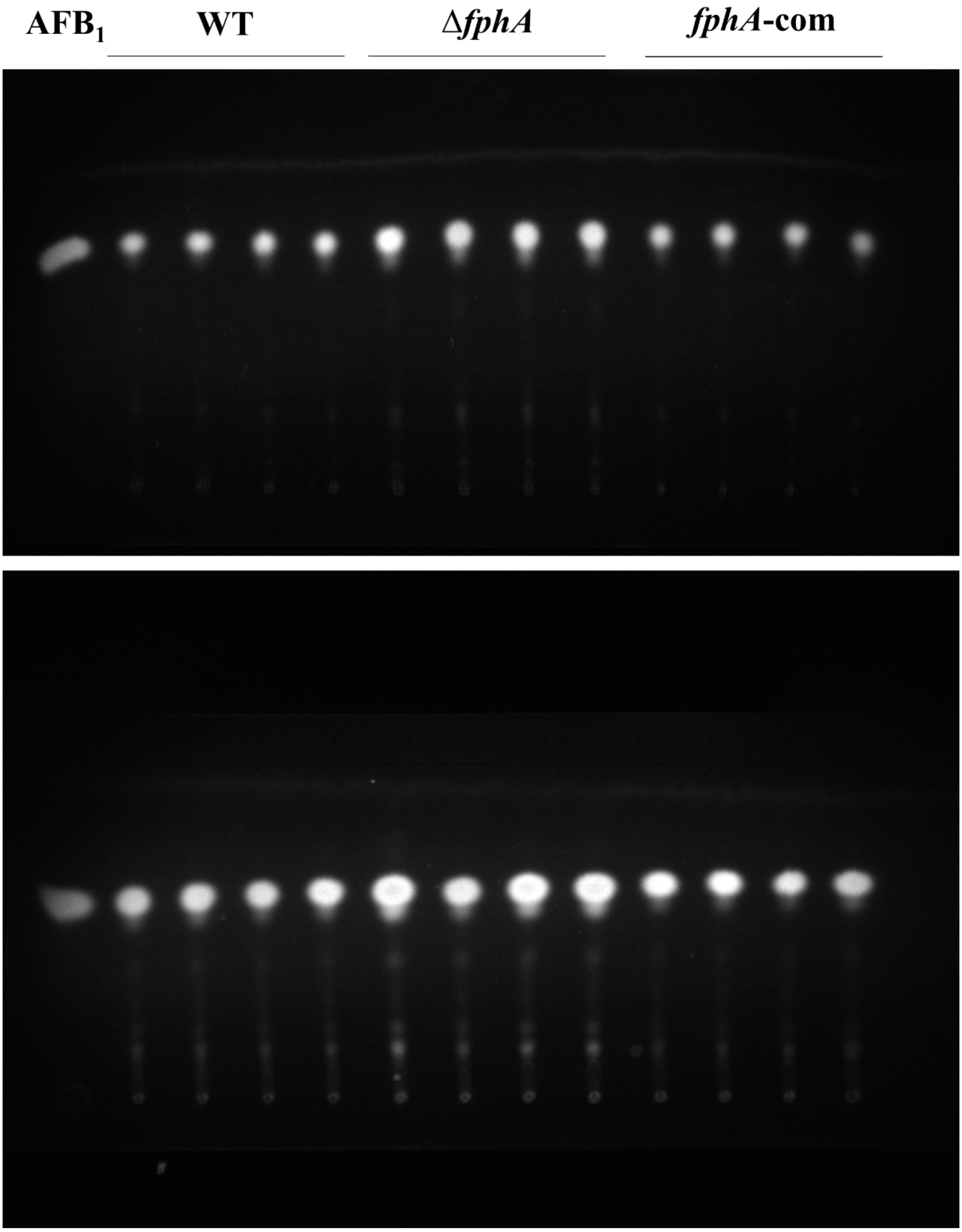

Supplementary Figure S3. The whole image of TLC plate for analysis of aflatoxins synthesized in WT, Δ*fphA* and *fphA*-com strains. The upper panel represents the TLC plate for aflatoxins from *A. flavus* in the dark, and the lower panel represents the TLC plate for aflatoxins from *A. flavus* in red light.

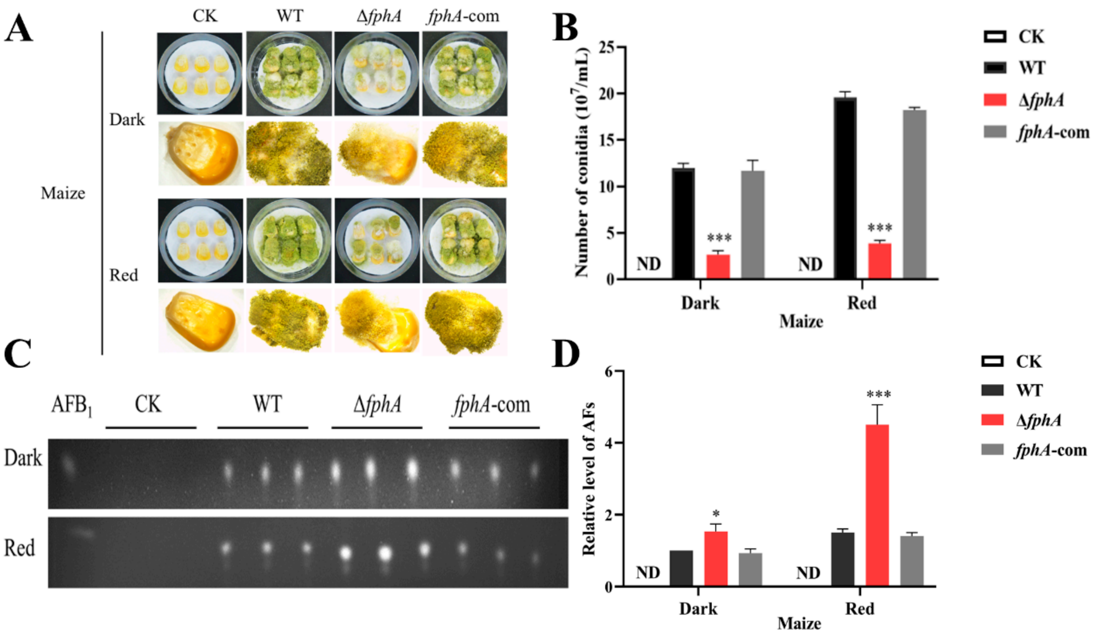

Supplementary Figure S4. The effect of FphA on pathogenicity of *A. flavus*. (A) The morphology of maize infected by WT, Δ*fphA* and *fphA*-com of *A. flavus*. (B) The comparison of conidia number in maize infected with WT, Δ*fphA* and *fphA*-com strains. (C) TLC chromatography of aflatoxins in maize infected with WT, Δ*fphA* and *fphA*-com strains. (D) AF levels from maize infected with WT, Δ*fphA* and *fphA*-com as in C. ND represents not detectable. (*, $p<0.05$; ***, $p<0.001$).
